# Monoamine Oxidase-A Inhibition and Associated Antioxidant Activity in Plant Extracts with Potential Antidepressant Actions

**DOI:** 10.1155/2018/4810394

**Published:** 2018-01-15

**Authors:** Tomás Herraiz, Hugo Guillén

**Affiliations:** Instituto de Ciencia y Tecnología de Alimentos y Nutrición (ICTAN), Spanish National Research Council (CSIC), Juan de la Cierva 3, 28006 Madrid, Spain

## Abstract

Monoamine oxidase (MAO) catalyzes the oxidative deamination of amines and neurotransmitters and is involved in mood disorders, depression, oxidative stress, and adverse pharmacological reactions. This work studies the inhibition of human MAO-A by* Hypericum perforatum*,* Peganum harmala, *and* Lepidium meyenii, *which are reported to improve and affect mood and mental conditions. Subsequently, the antioxidant activity associated with the inhibition of MAO is determined in plant extracts for the first time.* H. perforatum* inhibited human MAO-A, and extracts from flowers gave the highest inhibition (IC_50_ of 63.6 *μ*g/mL). Plant extracts were analyzed by HPLC-DAD-MS and contained pseudohypericin, hypericin, hyperforin, adhyperforin, hyperfirin, and flavonoids. Hyperforin did not inhibit human MAO-A and hypericin was a poor inhibitor of this isoenzyme. Quercetin and flavonoids significantly contributed to MAO-A inhibition.* P. harmala *seed extracts highly inhibited MAO-A (IC_50_ of 49.9 *μ*g/L), being a thousand times more potent than* H. perforatum* extracts owing to its content of *β*-carboline alkaloids (harmaline and harmine).* L. meyenii* root (maca) extracts did not inhibit MAO-A. These plants may exert protective actions related to antioxidant effects. Results in this work show that* P. harmala* and* H. perforatum *extracts exhibit antioxidant activity associated with the inhibition of MAO (i.e., lower production of H_2_O_2_).

## 1. Introduction

The enzyme monoamine oxidase (MAO) metabolizes xenobiotic and endogenous amines and neurotransmitters including serotonin, dopamine, norepinephrine, tyramine, tryptamine, and the neurotoxin MPTP [1, 2]. It occurs as two isoenzymes, MAO-A and MAO-B, which play an important role in the central nervous system (CNS) and peripheral organs. MAO-B is involved in neurodegenerative diseases and MAO-A in psychiatric conditions and depression. Inhibitors of MAO-B are useful as neuroprotectants, whereas inhibitors of MAO-A are effective antidepressants although their use may trigger adverse reactions (e.g., hypertensive crisis with foods containing tyramine) [1]. On the other hand, the oxidation of biogenic amines and neurotransmitters by MAO enzymes generates hydrogen peroxide (H_2_O_2_), oxygen radicals, and aldehydes, which are risk factors for cell oxidative injury. Therefore, the inhibition of MAO may result in protection against oxidative stress and neurotoxins [1, 3, 4]. Recent investigations have pointed out that plant and food extracts may inhibit MAO enzymes resulting in the above-mentioned biological effects [3, 5–14]. On the other hand, as a result of MAO inhibition, those products might be involved in undesirable interactions with other herbal preparations, foods, or drugs [1].

*Hypericum perforatum* L. (family Hypericaceae) (St. John's wort) is widely used for health purposes and their products are commercially available as herbs, nutraceuticals, teas, tinctures, juices, oily macerates, phytopharmaceuticals, and food additives and supplements [15, 16].* H. perforatum *is popular for treatment of mild and moderate depression [17–19]. It may trigger adverse pharmacological interactions with others herbs, drugs, or foods [20–22]. Its ability to alleviate and improve mood disorders and depression is attributed to active compounds that exhibit antidepressant properties [23, 24]. The most accepted mechanism of action is monoamine reuptake inhibition but additional mechanisms including monoamine oxidase inhibition and synergistic effects can be involved [17].* Peganum harmala* (family Zygophyllaceae) and* Lepidium meyenii *(family Brassicaceae) (maca) are plants with CNS effects and potential antidepressant actions [14, 25, 26].* P. harmala, *native from the Mediterranean region and Asia and extended to North America areas, is used as a multipurpose health remedy including CNS disorders. Preparations of this plant may trigger adverse pharmacological interactions [27].* L. meyenii* is an edible plant from the central Andes whose roots are used as a food energizer and nutraceutical to improve physical and mental conditions and fertility [28]. The purpose of this work was to study the inhibition of human MAO-A by extracts of* H. perforatum, P. harmala, *and* L. meyenii* (maca) as well as by their active components that were identified and analyzed by HPLC-DAD-MS and subsequently evaluate the antioxidant activity which is specifically associated with the inhibition of MAO. This specific antioxidant activity is determined for the first time in plant extracts.

## 2. Materials and Methods

*Hypericum perforatum* L. plants collected in Ciudad Real (Spain) were dried and separated in parts: flowers; top aerial portions of the plant including branched stems and leaves but no flowers; and main stems (central and lower) and roots. They were ground and the powder used for sample preparation. Commercial herbs and herbal supplements (capsules and tablets) of* H. perforatum* were also purchased in local herbal shops.* Peganum harmala* L. plant and seeds were collected in Toledo (Spain).* Lepidium meyenii* (maca) both as powder and commercial tablets were obtained from Peru and local shops. Hypericin standard (>95% purity by HPLC) from HWI Analytik GMBH pharma solutions, hyperforin dicyclohexylammonium salt, quercetin, harmaline, harmine, catalase, clorgyline, 3,3′,5,5′-tetramethylbenzidine (TMB), and horseradish peroxidase (HRP) type II were purchased from Sigma-Aldrich.

### 2.1. Sample Preparation of Plant Extracts

Samples containing* H. perforatum* (i.e., plant parts, herbal preparation, capsules, or tablets) (500 mg) were homogenized in 10 mL of water/methanol (1 : 1) by using an Ultra Turrax homogenizer, centrifuged at 10000 rpm for 10 min, and the supernatant was collected. The process was repeated twice with the residue and the three supernatant fractions collected, mixed and analyzed by HPLC as mentioned below. After three consecutive extractions, the recoveries of hypericin and pseudohypericin were higher than 97%. Samples of* L. meyenii* (maca) (500 mg) and* P. harmala* seeds (500 mg) were homogenized, respectively, in 10 mL of water/methanol (1 : 1) or 10 mL of 0.6 M perchloric acid : methanol (1 : 1) by using an Ultra Turrax homogenizer, centrifuged at 10000 rpm for 10 min, and the supernatant was collected. This process was repeated twice with the residue and the collected supernatants were mixed and analyzed by HPLC as mentioned below.

### 2.2. RP-HPLC Analysis of Plant Extracts

The analysis of* H. perforatum* extracts was performed by RP-HPLC with UV diode array and fluorescence detection using a HPLC 1050 (Agilent) coupled with a 1100 diode array detector (DAD) (Agilent) and a 1046A-fluorescence detector. A 150 × 3.9 mm* i.d.*, 4 *μ*m, Nova-pak C18 column (Waters) was used for separation. Chromatographic conditions were 50 mM ammonium phosphate buffer (pH 3) (buffer A) and 20% of A in acetonitrile (buffer B). The gradient was programmed from 0% (100% A) to 32% B in 8 min and 100% B at 10 min. The flow rate was 1 mL/min, the column temperature was 40°C, and the injection volume was 20 *μ*L. Detection of hypericins was carried out by absorbance at 590 nm and fluorescence at 236 nm for excitation and 592 nm for emission. The concentration of hypericin was determined from a calibration curve of response (absorbance at 590 nm) versus concentration with solutions made in the laboratory from hypericin standard. The same response factor was applied to pseudohypericin, protohypericin, and protopseudohypericin. Flavonoids and flavonoid glycosides were analyzed at 265 nm and 355 nm and the concentration of quercetin was determined at 355 nm from a calibration curve of response versus concentration. The HPLC fraction corresponding to flavonoids and flavonoid glycosides (7 to 11 min) was collected by successive injections of* H. perforatum *extract (herbs) and, after evaporation in vacuum, dissolved in 30% methanol and used for MAO-A inhibition. The phloroglucinols (hyperforin, adhyperforin, hyperfirin, and adhyperfirin) were analyzed at 280 nm by using the same column (Nova-pak C18) and conditions but under isocratic elution with 20% of 50 mM ammonium phosphate buffer, pH 3, and 80% of acetonitrile. The concentration of these compounds was determined from a calibration curve of hyperforin standard. The analysis of *β*-carboline alkaloids in* P. harmala* and* L. meyenii* was carried out as previously described [14, 29].

### 2.3. Identification by HPLC-ESI-Mass Spectrometry

Identification of compounds in* H. perforatum* extracts was done by HPLC-MS (electrospray-negative ion mode) by using a 1200 series HPLC-DAD coupled to a 6110 quadrupole-MS (Agilent). Chromatographic separation was performed on a 150 × 2.1 mm* i.d.* Zorbax SB-C18 (5 *μ*m) column (Agilent Technologies). The chromatographic conditions were eluent A: formic acid (0.1%); B: formic acid (0.1%) in acetonitrile; gradient: 0% to 70% B in 8 min and 100% B at 10 min, flow rate: 0.3 mL/min; *T*: 40°C; mass range: 50–700 u, and cone voltage: 150 V. For identification of phloroglucinols (e.g., hyperforin), separation was done using a Nova-pak C18 (4 *μ*m) column with the same eluents and isocratic elution (eluent A, 20% and eluent B, 80%) at a flow rate of 0.7 mL/min and mass spectra recorded in negative and positive ionization. Identification of compounds was done on the basis of mass spectra, UV-vis spectra (DAD) of chromatographic peaks, and coelution with standards. *β*-Carbolines in* P. harmala* and* L. meyenii* were identified as previously described [14, 29].

### 2.4. Monoamine Oxidase (MAO-A) Inhibition Assays

MAO assays were performed as elsewhere [8, 11, 14]. Briefly, membrane protein fractions containing MAO-A (BD-Gentest) were diluted to the desired concentrations in 100 mM potassium phosphate buffer (pH 7.4). A 0.2 mL reaction mixture containing 0.01 mg/mL protein and 0.25 mM kynuramine in 100 mM potassium phosphate (pH 7.4) was incubated at 37°C for 40 min. After incubation, the reaction was stopped by the addition of 2 N NaOH (75 *μ*L), followed by the addition of 70% HClO_4_ (25 *μ*L), and the sample was centrifuged (10000*g*) for 10 min. The supernatant (20 *μ*L) was injected into the HPLC and the deamination product of kynuramine (i.e., 4-hydroxyquinoline) formed during enzymatic reaction determined by RP-HPLC-diode array detection at 320 nm. A response curve of area versus concentration was constructed to calculate the concentration of 4-hydroxyquinoline. In order to perform assays of MAO inhibition, aliquots of extracts from plants or commercial preparations or instead pure compounds were conveniently diluted and added to reaction mixtures containing kynuramine (0.25 mM) and MAO-A (0.01 mg/mL protein) in 100 mM potassium phosphate buffer (pH 7.4), with enzymatic reaction and analysis carried out as above, and compared with the corresponding controls containing solvent. The standard inhibitor clorgyline was used as a positive control for inhibition (>90% inhibition at 2.5 *μ*M). Incubations were carried out at least in duplicate from different experiments and the IC_50_ values were calculated using GraphPad Prism 4.0.

### 2.5. Determination of Antioxidant Activity Associated with Monoamine Oxidase (MAO) Inhibition

Assays (0.2 mL) of reaction mixtures in 70 mM potassium phosphate buffer (pH 7.4), containing 0.025 mg/mL MAO-A protein and 0.25 mM kynuramine, were incubated at 37°C for 40 min in the absence (control assays) or in the presence of plant extracts. MAO assays were also performed in presence of clorgyline (25 *μ*M), a classical inhibitor of MAO-A (positive control of inhibition), or catalase enzyme (100 *μ*g/mL). After the incubation period, the reaction mixture was added with activated charcoal (3.5 mg), mixed, and filtered (0.45 *μ*m). The solution was added with 20 *μ*L of 10 mM tetramethylbenzidine (TMB) in 40% DMSO and 20 *μ*L of horseradish peroxidase (HRP) type II (1 mg/mL), kept 5 min, and added with 0.3 mL of 0.5 M H_2_SO_4_ solution. The absorbance at 450 nm was measured to determine TMB diimine, a yellow product resulting from the oxidation of TMB by HRP and the H_2_O_2_ generated in the oxidative deamination catalyzed by MAO. The oxidation of TMB in the presence of inhibitors of MAO was compared with the corresponding controls without inhibitors and appropriate blanks showed absence of interferences.

## 3. Results and Discussion

**Figure 1:**
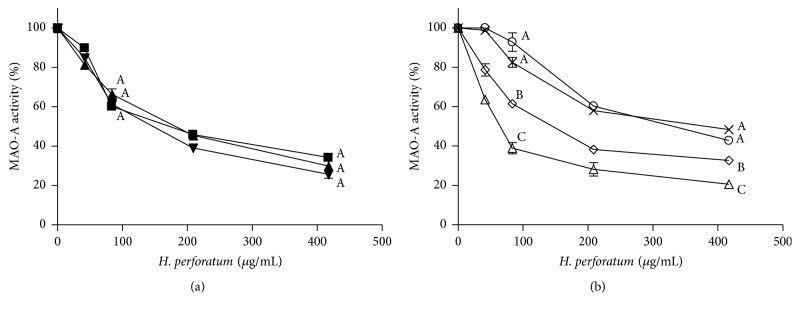
Inhibition of human monoamine oxidase-A (MAO-A) by extracts of commercial preparations* of H. perforatum* (a) (capsules, ■; tablets, ▲; herbs, ▼), and extracts from different parts of the plant (b) (flowers, ∆; top stems, ◊; main stems (central), ×; roots, ○). Significant differences (*p* < 0.05) among extracts at a selected concentration are indicated with different letters.

**Figure 2:**
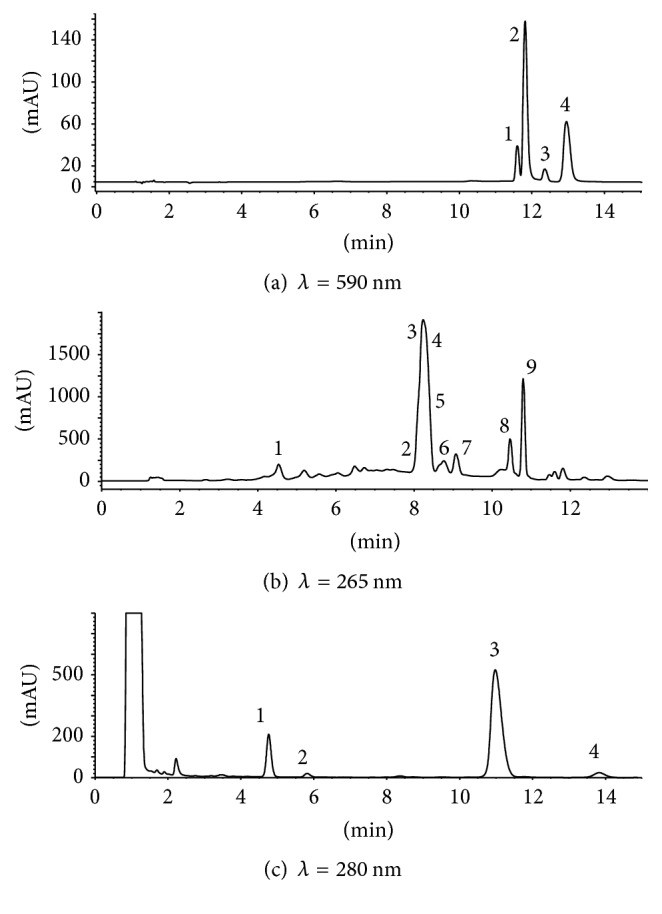
HPLC chromatograms of extracts from* H. perforatum* flowers. (a) Detection of hypericins at 590 nm.** 1**: protopseudohypericin;** 2**: pseudohypericin;** 3**: protohypericin; and** 4**: hypericin. (b) Detection of phenols and flavonoids at 265 nm.** 1**: chlorogenic acid;** 2**: rutin;** 3**: hyperoside;** 4**: isoquercitrin;** 5**: miquelianin;** 6**: acetyl hyperoside;** 7**: quercitrin;** 8**: quercetin; and** 9**: biapigenin. (c) Detection of phloroglucinols at 280 nm.** 1**: hyperfirin,** 2**: adhyperfirin;** 3**: hyperforin; and** 4**: adhyperforin.

**Figure 3:**
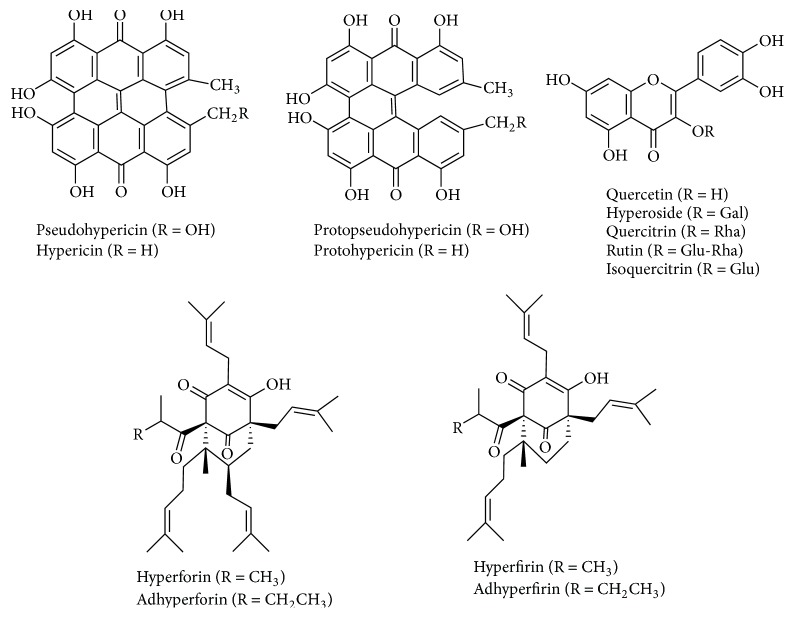
Structures of compounds identified in* H. perforatum*: hypericins, quercetin, and quercetin flavonoids and phloroglucinols (hyperforin, adhyperforin, hyperfirin, and adhyperfirin).

Commercial preparations of* H. perforatum* inhibited human MAO-A with similar potency: IC_50_ values of 142.3 ± 30.6 *μ*g/mL (herbal preparation), 193 ± 61 *μ*g/mL (capsules), and 173 ± 29 *μ*g/mL (tablets) (Figure 1(a)). Regarding plants,* H. perforatum* extracts from flowers afforded the highest inhibition (IC_50_ of 63.6 ± 9.4 *μ*g/mL) followed by aerial stems and leaves (IC_50_ 143.6 ± 16.5 *μ*g/mL), and the lowest in root extracts (Figure 1(b)). Extracts from the aerial parts of* H. perforatum* were analyzed by HPLC-DAD-ESI (electrospray-negative ionization). They showed the presence of two major naphthodianthrones identified as pseudohypericin and hypericin (Figure 2(a) and Table 1). Flower extracts had two additional compounds identified as protopseudohypericin and protohypericin. Phenolics and flavonoids abounded in* H. perforatum* extracts (Figure 2(b)). Chlorogenic acid and the quercetin glycosides rutin, hyperoside, isoquercitrin, miquelianin, acetyl hyperoside, and quercitrin, as well as free quercetin and biapigenin, were identified by HPLC-DAD (ESI negative ionization) and DAD (Table 1). On the other hand, flower extracts contained four phloroglucinols (Figure 2(c)) that were identified by HPLC-DAD-MS (ESI negative and positive ionization) and DAD as hyperforin, adhyperforin, hyperfirin, and adhyperfirin (Table 1). The presence of these compounds (Figure 3) in the plant agrees with other results [15, 30, 31]. The content of the main components was determined by HPLC (Table 2). Concentration of pseudohypericin was higher than hypericin, whereas protopseudohypericin and protohypericin were minor compounds (0.4 *μ*g/mg of protopseudohypericin and 0.17 *µ*g/mg of protohypericin were detected in flowers). In the plant, the highest content of hypericins was found in flowers with significantly low levels detected in stems and absence in roots. Hyperforin was highly abundant in flowers (27.2 *μ*g/mg), whereas the concentration in commercial preparations ranged from 0.36 to 2.4 *μ*g/mg. In flowers, adhyperforin (1.4 ± 0.07 *μ*g/mg), hyperfirin (4.2 ± 0.02 *μ*g/mg), and adhyperfirin (0.46 ± 0.02 *μ*g/mg) also appeared. Flavonoids abounded in* H. perforatum* and most of them were quercetin glycosides (Figure 2(b)) whose presence was significantly higher in flowers than in other parts of the plant. The content of free quercetin in flowers was 2.0 *μ*g/mg, whereas a content of 6.7 *μ*g/mg was determined in commercial preparations.

**Figure 4:**
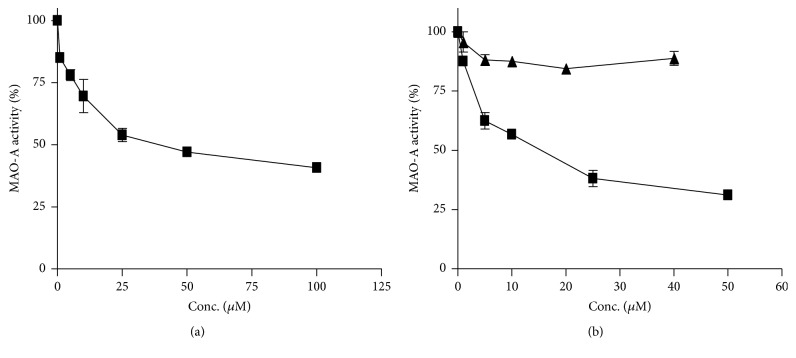
Inhibition of human monoamine oxidase-A (MAO-A) by active components of* H. perforatum*: hypericin (a) and quercetin (■) and hyperforin (▲) (b).

The inhibition of MAO-A by* H. perforatum *extracts indicates occurrence of inhibitors. Hypericins, hyperforin, and flavonoids are possible contributors to this inhibition and were evaluated as inhibitors (Figure 4). Hypericin inhibited MAO-A (IC_50_ of 35.5 ± 2.1 *μ*M or 17.9 *μ*g/mL) (Figure 4(a)). From the concentration in Table 2, hypericin is a weak contributor to MAO inhibition in* H. perforatum *extracts. Indeed, the calculated content of hypericin at IC_50_ value in assays of flower extract (i.e., 63.6 *μ*g/mL) was 0.1 *μ*g/mL which is low compared with IC_50_ of hypericin (17.9 *μ*g/mL). Hyperforin did not inhibit MAO-A (Figure 4(b)). Quercetin inhibited human MAO-A (Figure 4(b)) with an IC_50_ value of 11.1 ± 0.8 *μ*M (i.e., 3.36 *μ*g/mL). Then, quercetin was a better inhibitor than hypericin although its potency was still low to explain entire inhibition of extracts. Thus, the calculated content of quercetin at IC_50_ in assays of flower extract was 0.13 *μ*g/mL which is lower than the IC_50_ of quercetin (3.4 *μ*g/mL). When the fraction corresponding to quercetin glycosides and flavonoids (7–11 min, Figure 2(b)) was collected by RP-HPLC, it inhibited MAO-A (90% inhibition at 700 *μ*g/mL extract) indicating a contribution of these compounds to MAO inhibition in* H. perforatum*, probably by additive effects. Then, inhibition of MAO-A could arise from components such as quercetin and related flavonoids (i.e., quercetin glycosides) which are abundant in the plant. In addition, minor compounds not identified here could also contribute to MAO inhibition as major compounds in Table 2 do not explain whole inhibition.

**Figure 5:**
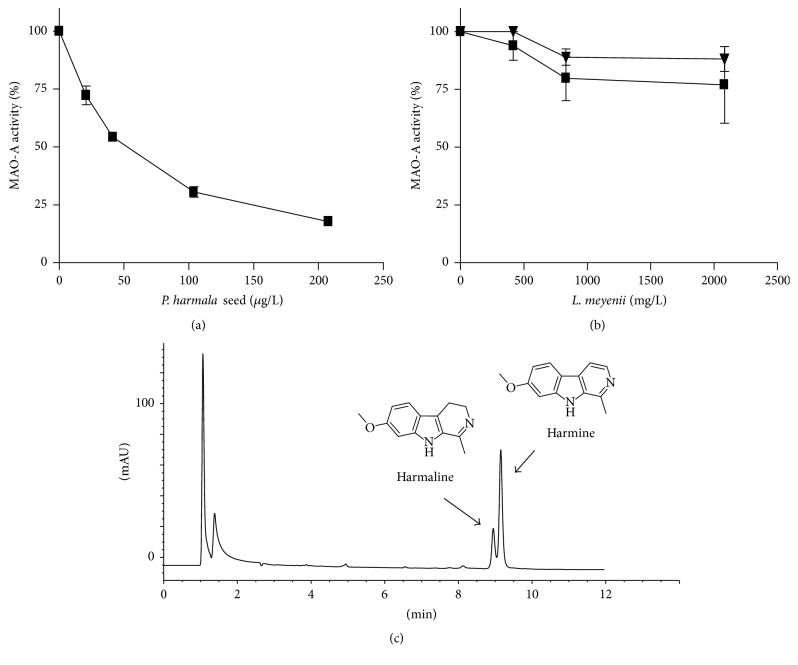
Inhibition of human monoamine oxidase-A (MAO-A) by* P. harmala* seed (a) and* L. meyenii* root (maca) extracts (b) (capsules, ▼ and powder, ■). (c) HPLC chromatogram of* P. harmala* seed extract which potently inhibited human MAO-A. Absorbance detection at 254 nm. Compounds identified are harmaline (*m/z* at 215 (M + H)^+^, UV_max_ at 375 nm) and harmine (*m/z* at 213 (M + H)^+^, UV_max_ at 245 and 322 nm).

Extracts from* P. harmala* seeds highly inhibited human MAO-A (Figure 5(a)) affording an IC_50_ value of 49.9 ± 5.6 *μ*g/L. Chromatographic analysis indicated that inhibition was due to the presence of the *β*-carboline alkaloids, harmaline and harmine, that were identified by HPLC-DAD-MS (Figure 5(c)). The content of these alkaloids determined in seeds was 48.5 mg/g for harmaline and 40.0 mg/g for harmine (this means 2.4 ng/mL and 2.0 ng/mL, resp., into assays at the IC_50_). Therefore, the inhibition potency of MAO-A by* P. harmala* seeds was 1274 times more potent than that of* H. perforatum* flowers. As shown in Figure 5(b),* Lepidium meyenii* root extracts did not inhibit human MAO-A.* L. meyenii *(maca) is a popular plant from the Andes highlands whose roots are increasingly used for its nutritional and medicinal properties as energizing and to improve mood and sexual performance [28, 32]. Previous reports have indicated that they contain alkaloids including *β*-carbolines [25, 26] that might inhibit MAO. Analysis of extracts for *β*-carboline alkaloids gave 25 *μ*g/g (maca powder) and 11.7 *μ*g/g (capsules) of 1-methyl-1,2,3,4-tetrahydro-*β*-carboline-3-carboxylic acid as a major compound. This specific *β*-carboline is not an inhibitor of MAO-A [8, 11].

**Figure 6:**
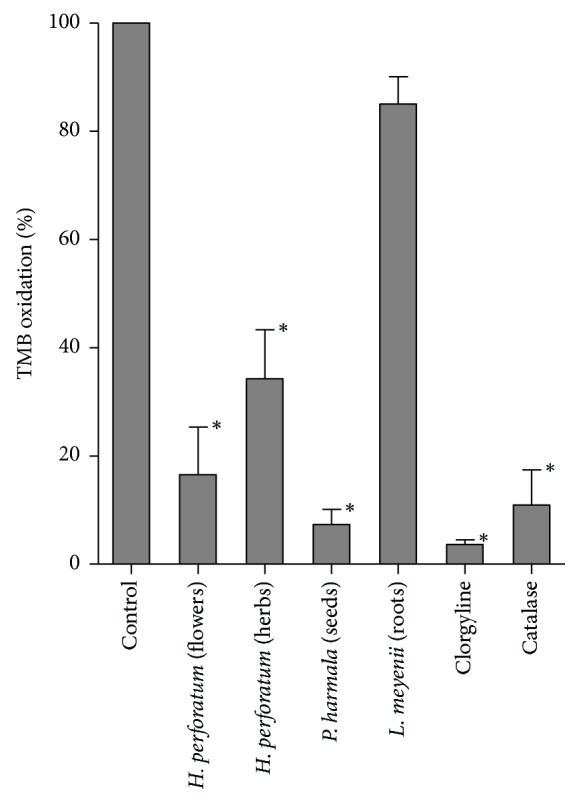
Antioxidant activity associated with MAO inhibition in assays coupling activity of MAO-A with the subsequent oxidation of tetramethylbenzidine (TMB) in the presence H_2_O_2_ generated in the reaction of MAO, and horseradish peroxidase (HRP). The graph shows the oxidation of TMB to diimine (absorbance at 450 nm) in control assays (100%), and in the presence of* H. perforatum* (herbs and flower extracts, 800 *μ*g/mL),* P. harmala* seed extracts (0.8 *μ*g/mL),* L. meyenii* root (maca) extracts (800 *μ*g/mL), clorgyline (a standard inhibitor of MAO-A) (25 *μ*M), and catalase (100 *μ*g/mL). ^*∗*^Significant differences (*p* < 0.01) compared to controls.

MAO generates hydrogen peroxide (H_2_O_2_) that is involved in oxidative cell damage and pathological conditions [1, 3, 4, 33–36]. Then, the inhibition of MAO may result in specific antioxidant actions [37]. In order to study the antioxidant activity associated with MAO inhibition, experiments were designed in this research which linked the activity of MAO-A with the oxidation of tetramethylbenzidine (TMB) by horseradish peroxidase (HRP) and the H_2_O_2_ produced during oxidative deamination catalyzed by MAO (Figure 6).* H. perforatum* and* P. harmala* extracts which inhibited MAO-A as shown above highly decreased oxidation of TMB. In contrast,* L. meyenii* root (maca) extracts that did not inhibit MAO had a low antioxidant activity in this assay. Clorgyline which is a potent inhibitor of MAO-A highly decreased the oxidation of TMB when used as a control. The same happened with the presence of catalase in the media that removes H_2_O_2_ generated by MAO-A. Therefore, these results indicate that* H. perforatum *and* P. harmala* extracts afforded specific antioxidant actions associated with a lower production of H_2_O_2_ by inhibition of MAO.

*H. perforatum* improves mood disorders and depression [17, 18, 38]. As shown here, it contains compounds such as hyperforin, hypericins, and flavonoids responsible for antidepressant effects (Figure 2 and Table 2). However, the specific mechanism for antidepressant action is not completely understood. The most accepted mechanism is inhibition of monoamine reuptake [23, 24, 39, 40]. However, some studies suggest a combination of mechanisms and synergistic effects [17, 41].* P. harmala* exerts numerous biological and pharmacological actions. Their seeds are increasingly used for recreational purposes owing to their psychoactive and neuroactive effects [14]. The inhibition of human MAO-A is an established mechanism for antidepressant action [1]. Both irreversible and reversible inhibitors of MAO-A (*e.g*., phenelzine and moclobemide) are successfully used as antidepressants. In this study,* H. perforatum* extracts inhibited human MAO-A. However, this inhibition was moderate. It was more than one thousand times lower than that of* P. harmala *seed extracts. Sacher et al. have reported that the occupancy of MAO-A sites into the human brain determined by PET imaging with ^11^C-harmine binding (i.e., the same *β*-carboline responsible for MAO inhibition in* P. harmala*) was high for a reversible inhibitor of MAO such as moclobemide but low for* H. perforatum* extract (St. John's wort) [42]. This means that the inhibitors of MAO-A in* H. perforatum* do not bind efficiently to active sites of MAO-A in the brain in contrast to the *β*-carboline harmine. The inhibitors of MAO-A in* H. perforatum* are flavonoids such as quercetin and their glycosides and the levels of these compounds that reach the brain might not be enough to occupy the sites of MAO-A in the brain and inhibit the enzyme [43]. In contrast, the inhibitors of* P. harmala* are *β*-carboline alkaloids including harmine and harmaline which have a very good brain penetration, bind with high affinity to MAO sites, and exhibit antidepressant effects [44–46]. Therefore,* P. harmala* could afford antidepressant effects by MAO inhibition. In this regard, it could be of interest to investigate the antidepressant effects of* H. perforatum* and* P. harmala* alone and in combination as they have different mechanisms of action.

The inhibition of MAO-A by* H. perforatum* and* P. harmala* extracts may contribute to other biological effects of these plants such as antioxidant actions and adverse pharmacological reactions. Extracts of these plants exert neuroprotective and anti-inflammatory effects which have been related to antioxidant activity [6, 9, 47–50]. In this regard by using a new procedure, results in this work have evidenced that* H. perforatum *and* P. harmala* extracts show antioxidant activity associated with the inhibition of MAO (lower production of H_2_O_2_). On the other hand, one of the major limitations to the use of these plants is their potential for producing adverse interactions with other herbs, foods, and drugs [17, 20, 21, 27]. The inhibition of MAO-A may trigger adverse effects under certain circumstances [1, 14].

## 4. Conclusions

Extracts from* H. perforatum* inhibited human MAO-A, and extracts from flowers were the most potent inhibitors. They were studied by HPLC-DAD-MS and contained pseudohypericin, hypericin, hyperforin, adhyperforin, hyperfirin, and flavonoids. The highest content of these compounds appeared in flowers. Hypericin was a weak inhibitor of MAO-A; hyperforin did not inhibit the enzyme and quercetin was a moderate inhibitor. The fraction of quercetin glycosides and flavonoids contributed to MAO inhibition.* P. harmala* seed extracts highly inhibited MAO-A and its potency of inhibition was more than a thousand times higher than* H. perforatum* extracts owing to its content in harmaline and harmine alkaloids.* L. meyenii* root (maca) extracts did not inhibit MAO-A. The inhibition of MAO-A may not explain the entire CNS effects attributed to* H. perforatum *but it is expected to contribute to these actions in* P. harmala.* These plants exert antioxidant effects. By using a new method this work have evidenced that* P. harmala* and* H. perforatum *extracts exhibit antioxidant activity associated with the inhibition of MAO.

## Figures and Tables

**Table 1:** Compounds identified in *H. perforatum.*

Compounds	ESI-neg. ion (M − H)^−^	UV max (DAD)
*Naphthodianthrones*		
Pseudohypericin	519	547, 590
Hypericin	503	547, 590
Protopseudohypericin	521	370, 539
Protohypericin	505	370, 539
*Phenolic comp.*		
Chlorogenic acid	353	324
Rutin	609	256, 355
Hyperoside	463	256, 355
Isoquercitrin	463	256, 355
Miquelianin	477	256, 355
Acetyl hyperoside	505	263, 352
Quercitrin	447	255, 348
Quercetin	301	255, 369
Biapigenin	537	268, 331
*Phloroglucinols*		
Hyperfirin^a^	467	274
Adhyperfirin^a^	481	274
Hyperforin^a^	535	274
Adhyperforin^a^	549	274

^a^These compounds gave also their corresponding (M + H)^+^ and (M + K)^+^ ions under ESI-positive ionization.

**Table 2:** Content (*μ*g/mg)^1^ of the main active components in *H. perforatum* samples.

*H. perforatum* samples	Pseudohypericin	Hypericin	Hyperforin	Quercetin
Plant				
Stems (top)	0.25 ± 0.03^a^	0.11 ± 0.01^a^	1.48 ± 0.3^a^	0.28 ± 0.12^a^
Stems (central)	0.1 ± 0.04^a^	0.04 ± 0.01^a^	0.59 ± 0.16^a^	0.19 ± 0.01^a^
Roots	-	-	0.77 ± 0.1^a^	-
Flowers	2.78 ± 0.7^b^	1.58 ± 0.31^b^	27.2 ± 0.6^b^	2.04 ± 0.08^b^
Commercial prep.				
Herbs	0.51 ± 0.05^a^	0.11 ± 0.01^a^	1.18 ± 0.03^a^	0.71 ± 0.4^a^
Capsules	2.41 ± 0.2^b^	0.83 ± 0.1^b^	2.42 ± 0.01^b^	2.4 ± 0.9^a^
Tablets	2.39 ± 0.2^b^	2.11 ± 0.2^c^	0.36 ± 0.1^c^	6.7 ± 1.7^b^

Significant differences (*p* < 0.05) for a compound within a group are indicated with different letters. ^1^*μ*g of compound/mg of plant tissue for plants parts and herbs or mg of powder in capsules and tablets.

## References

[1] Youdim M. B. H., Edmondson D., Tipton K. F. (2006). The therapeutic potential of monoamine oxidase inhibitors. *Nature Reviews Neuroscience*.

[2] Herraiz T., Guillén H., Galisteo J. (2013). Metabolite Profile Resulting from the Activation/Inactivation of 1-Methyl-4-phenyl-1,2,3,6-tetrahydropyridine and 2-Methyltetrahydro-*β*-carboline by Oxidative Enzymes. *BioMed Research International*.

[3] Herraiz T., Guillén H. (2011). Inhibition of the bioactivation of the neurotoxin MPTP by antioxidants, redox agents and monoamine oxidase inhibitors. *Food and Chemical Toxicology*.

[4] Cohen G., Kesler N. (1999). Monoamine oxidase and mitochondrial respiration. *Journal of Neurochemistry*.

[5] Farahani M. S., Bahramsoltani R., Farzaei M. H., Abdollahi M., Rahimi R. (2015). Plant-derived natural medicines for the management of depression: An overview of mechanisms of action. *Reviews in the Neurosciences*.

[6] Gómez del Rio M. A., Sánchez-Reus M. I., Iglesias I. (2013). Neuroprotective properties of standardized extracts of hypericum perforatum on rotenone model of parkinson's disease. *CNS and Neurological Disorders - Drug Targets*.

[7] Herraiz T. (2007). Identification and occurrence of *β*-carboline alkaloids in raisins and inhibition of monoamine oxidase (MAO). *Journal of Agricultural and Food Chemistry*.

[8] Herraiz T., Chaparro C. (2006). Human monoamine oxidase enzyme inhibition by coffee and *β*-carbolines norharman and harman isolated from coffee. *Life Sciences*.

[9] Mohanasundari M., Sabesan M. (2007). Modulating effect of Hypericum perforatum extract on astrocytes in MPTP induced Parkinson's disease in mice. *European Review for Medical and Pharmacological Sciences*.

[10] Kennedy D. O., Wightman E. L. (2011). Herbal extracts and phytochemicals: plant secondary metabolites and the enhancement of human brain function. *Advances in Nutrition*.

[11] Herraiz T., Chaparro C. (2006). Analysis of monoamine oxidase enzymatic activity by reversed-phase high performance liquid chromatography and inhibition by *β*-carboline alkaloids occurring in foods and plants. *Journal of Chromatography A*.

[12] Jäger A. K., Gauguin B., Andersen J., Ersen A. A., Gudiksen L. (2013). Screening of plants used in Danish folk medicine to treat depression and anxiety for affinity to the serotonin transporter and inhibition of MAO-A. *Journal of Ethnopharmacology*.

[13] Bandaruk Y., Mukai R., Kawamura T., Nemoto H., Terao J. (2012). Evaluation of the inhibitory effects of quercetin-related flavonoids and tea catechins on the monoamine oxidase-A reaction in mouse brain mitochondria. *Journal of Agricultural and Food Chemistry*.

[14] Herraiz T., González D., Ancín-Azpilicueta C., Arán V. J., Guillén H. (2010). *β*-carboline alkaloids in Peganum harmala and inhibition of human monoamine oxidase (MAO). *Food and Chemical Toxicology*.

[15] Silva B. A., Ferreres F., Malva J. O., Dias A. C. P. (2005). Phytochemical and antioxidant characterization of Hypericum perforatum alcoholic extracts. *Food Chemistry*.

[16] Orhan I. E., Kartal M., Gülpinar A. R. (2013). Assessment of antimicrobial and antiprotozoal activity of the olive oil macerate samples of Hypericum perforatum and their LC-DAD-MS analyses. *Food Chemistry*.

[17] Russo E., Scicchitano F., Whalley B. J. (2014). Hypericum perforatum: Pharmacokinetic, mechanism of action, tolerability, and clinical drug-drug interactions. *Phytotherapy Research*.

[18] Kasper S., Caraci F., Forti B., Drago F., Aguglia E. (2010). Efficacy and tolerability of Hypericum extract for the treatment of mild to moderate depression. *European Neuropsychopharmacology*.

[19] Linde K., Kriston L., Rücker G. (2015). Efficacy and acceptability of pharmacological treatments for depressive disorders in primary care: Systematic review and network meta-analysis. *Annals of Family Medicine*.

[20] Borrelli F., Izzo A. A. (2009). Herb-drug interactions with St John's Wort (hypericum perforatum): an update on clinical observations. *The AAPS Journal*.

[21] Hoban C. L., Byard R. W., Musgrave I. F. (2015). A comparison of patterns of spontaneous adverse drug reaction reporting with St. John's Wort and fluoxetine during the period 2000-2013. *Clinical and Experimental Pharmacology and Physiology*.

[22] Davidson J. R. T., Gadde K. M., Fairbank J. A. (2002). Effect of Hypericum perforatum (St John's wort) in major depressive disorder—a randomized controlled trial. *The Journal of the American Medical Association*.

[23] Muller W. E., Singer A., Wonnemann M., Hafner U., Rolli M., Schafer C. (1998). Hyperforin represents the neurotransmitter reuptake inhibiting constituent of Hypericum extract. *Pharmacopsychiatry*.

[24] Paulke A., Noldner M., Schubert-Zslavecz M., Wurglics M. (2008). St. John's wort flavonols and their metabolites show antidepressant activity and accumulate in brain after multiple oral doses. *Pharmazie*.

[25] Wang Y., Wang Y., McNeil B., Harvey L. M. (2007). Maca: An Andean crop with multi-pharmacological functions. *Food Research International*.

[26] Piacente S., Carbone V., Plaza A., Zampelli A., Pizza C. (2002). Investigation of the tuber constituents of maca (Lepidium meyenii Walp.). *Journal of Agricultural and Food Chemistry*.

[27] Frison G., Favretto D., Zancanaro F., Fazzin G., Ferrara S. D. (2008). A case of *β*-carboline alkaloid intoxication following ingestion of Peganum harmala seed extract. *Forensic Science International*.

[28] Gonzales G. F., Gonzales C., Gonzales-Castañeda C. (2009). *Lepidium meyenii* (Maca): a plant from the highlands of Peru—from tradition to science. *Forschende Komplementärmedizin*.

[29] Herraiz T. (2000). Tetrahydro-beta-carboline-3-carboxylic acid compounds in fish and meat: Possible precursors of co-mutagenic beta-carbolines norharman and harman in cooked foods. *Food Additives and Contaminants*.

[30] Tolonen A., Hohtola A., Jalonen J. (2003). Fast high-performance liquid chromatographic analysis of naphthodianthrones and phloroglucinols from Hypericum perforatum extracts. *Phytochemical Analysis*.

[31] Tatsis E. C., Boeren S., Exarchou V., Troganis A. N., Vervoort J., Gerothanassis I. P. (2007). Identification of the major constituents of Hypericum perforatum by LC/SPE/NMR and/or LC/MS. *Phytochemistry*.

[32] Stone M., Ibarra A., Roller M., Zangara A., Stevenson E. (2009). A pilot investigation into the effect of maca supplementation on physical activity and sexual desire in sportsmen. *Journal of Ethnopharmacology*.

[33] Halliwell B., Gutteridge J. M. (1999). *Free radicals in biology and medicine*.

[34] Herraiz T., Galisteo J. (2014). Naturally-occurring tetrahydro-*β*-carboline alkaloids derived from tryptophan are oxidized to bioactive *β*-carboline alkaloids by heme peroxidases. *Biochemical and Biophysical Research Communications*.

[35] Kaludercic N., Mialet-Perez J., Paolocci N., Parini A., Di Lisa F. (2014). Monoamine oxidases as sources of oxidants in the heart. *Journal of Molecular and Cellular Cardiology*.

[36] Mallajosyula J. K., Kaur D., Chinta S. J. (2008). MAO-B elevation in mouse brain astrocytes results in Parkinson's pathology. *PLoS ONE*.

[37] Herraiz T., Flores A., Fernández L. (2018). Analysis of monoamine oxidase (MAO) enzymatic activity by high-performance liquid chromatography-diode array detection combined with an assay of oxidation with a peroxidase and its application to MAO inhibitors from foods and plants. *Journal of Chromatography B: Analytical Technologies in the Biomedical and Life Sciences*.

[38] Bukhari I. A., Dar A. (2013). Behavioral profile of Hypericum perforatum (St. John's Wort) extract. A comparison with standard antidepressants in animal models of depression. *European Review for Medical and Pharmacological Sciences*.

[39] Zhai X.-J., Chen F., Chen C., Zhu C.-R., Lu Y.-N. (2015). LC-MS/MS based studies on the anti-depressant effect of hypericin in the chronic unpredictable mild stress rat model. *Journal of Ethnopharmacology*.

[40] Cott J. M. (1998). In vitro receptor binding and enzyme inhibition by an extract of Hypericum perforatum. *Psychopharmakotherapie*.

[41] Muller W. E., Rolli M., Schafer C., Hafner U. (1997). Effects of hypericum extract (LI 160) in biochemical models of antidepressant activity. *Pharmacopsychiatry*.

[42] Sacher J., Houle S., Parkes J. (2011). Monoamine oxidase A inhibitor occupancy during treatment of major depressive episodes with moclobemide or St. John's wort: an C-11 -harmine PET study. *Journal of Psychiatry and Neuroscience*.

[43] Yoshino S., Hara A., Sakakibara H. (2011). Effect of quercetin and glucuronide metabolites on the monoamine oxidase-A reaction in mouse brain mitochondria. *Nutrition*.

[44] Farzin D., Mansouri N. (2006). Antidepressant-like effect of harmane and other *β*-carbolines in the mouse forced swim test. *European Neuropsychopharmacology*.

[45] Herraiz T., Gilbert J., Senyuva H. Z. (2008). *β*-Carboline alkaloids. *Bioactive compounds in foods*.

[46] Herraiz T. (2016). N-methyltetrahydropyridines and pyridinium cations as toxins and comparison with naturally-occurring alkaloids. *Food and Chemical Toxicology*.

[47] Hammer K. D. P., Birt D. F. (2014). Evidence for contributions of interactions of constituents to the anti-inflammatory activity of hypericum perforatum. *Critical Reviews in Food Science and Nutrition*.

[48] Silva B. A., Malva J. O., Dias A. C. P. (2008). St. John's Wort (Hypericum perforatum) extracts and isolated phenolic compounds are effective antioxidants in several *in vitro* models of oxidative stress. *Food Chemistry*.

[49] Bensalem S., Soubhye J., Aldib I. (2014). Inhibition of myeloperoxidase activity by the alkaloids of *Peganum harmala* L. (Zygophyllaceae). *Journal of Ethnopharmacology*.

[50] Zou Y., Lu Y., Wei D. (2004). Antioxidant activity of a flavonoid-rich extract of Hypericum perforatum L. in vitro. *Journal of Agricultural and Food Chemistry*.

